# The Anticancer Peptide CIGB-552 Exerts Anti-Inflammatory and Anti-Angiogenic Effects through COMMD1

**DOI:** 10.3390/molecules26010152

**Published:** 2020-12-31

**Authors:** Hellen Daghero, Julio Raúl Fernández Massó, Soledad Astrada, Maribel Guerra Vallespí, Mariela Bollati-Fogolín

**Affiliations:** 1 Cell Biology Unit, Institut Pasteur Montevideo, Mataojo 2020, Montevideo 11400, Uruguay; hdaghero@pasteur.edu.uy (H.D.); soledad.astrada@gmail.com (S.A.); 2 Department of Genomic, Center for Genetic Engineering and Biotechnology, Cubanacan, P.O. Box 6162, Havana 10600, Cuba; julio.fernandez@cigb.edu.cu; 3 Institut d’Optique d’Aquitaine, Université de Bordeaux, 33400 Talence, France; 4 Pharmaceutical Department, Center for Genetic Engineering and Biotechnology, Cubanacan, P.O. Box 6162, Havana 10600, Cuba

**Keywords:** anti-cancer peptides, cell penetrating peptides, COMMD1, NF-kB, HIF

## Abstract

CIGB-552 is a synthetic anti-tumor peptide capable of reducing tumor size and increasing the lifespan of tumor-bearing mice. Part of its anti-cancer effects consists of inducing apoptosis, modulating NF-kB signaling pathway, and the angiogenesis process. Although one of its major mediators, the COMMD1 protein, has been identified, the mechanism by which CIGB-552 exerts such effects remains elusive. In the present study, we show the role of COMMD1 in CIGB-552 mechanism of action by generating the COMMD1 knock-out from the human lung cancer cell line NCI-H460. A microarray was performed to analyze both wild-type and KO cell lines with regard to CIGB-552 treatment. Additionally, different signaling pathways were studied in both cell lines to validate the results. Furthermore, the interaction between CIGB-552 and COMMD1 was analyzed by confocal microscopy. By signaling pathway analysis we found that genes involved in cell proliferation and apoptosis, oncogenic transformation, angiogenesis and inflammatory response are potentially regulated by the treatment with CIGB-552. We then demonstrated that CIGB-552 is capable of modulating NF-kB in both 2D and 3D cell culture models. Finally, we show that the ability of CIGB-552 to negatively modulate NF-kB and HIF-1 pathways is impaired in the COMMD1 knock-out NCI-H460 cell line, confirming that COMMD1 is essential for the peptide mechanism of action.

## 1. Introduction

CIGB-552 is an anticancer peptide developed from the screening of an Ala-library derived from de LALF_32–51_ region [1]. The original peptide LALF_32–51_ was not only able to bind and neutralize bacterial LPS but also modulate the inflammatory response mediated by LPS both in vitro and in vivo [2,3]. After an alanine scanning of the LALF_32–51_, the resulting peptides were evaluated by LPS-binding ability, antitumor effect and cell-penetration capacity in live cells, leading to a new peptide (named L-2) optimized for its anticancer activity [1]. In order to improve its pharmacokinetics properties, several modifications of the primary structure of L-2 led to CIGB-552, a second-generation anti-cancer peptide. CIGB-552 maintains the cell-penetrating capacity and shows a higher antitumor effect compared to LALF_32–51_ and L-2 [4,5]. The sequence of analogs peptides and their biological properties are shown in Table 1.

Apart from its cell-penetrating capacity, CIGB-552 has the ability to produce a cytotoxic effect by inducing apoptosis of cancer cell lines [1,4,7]. In particular, it has been shown that the cytotoxic effect of CIGB-552 depends on the presence of Copper Metabolism Mur 1 Domain containing protein 1 (COMMD1) [4]. CIGB-552 promotes the stabilization of the COMMD1 protein, a small protein involved in cellular copper homeostasis, ion transport, oxidative stress and the modulation of different transcription factors [4,8,9,10]. Among these, there are the nuclear factor kappa-light-chain-enhancer of activated B cells (NF-κB) and Hypoxia Induced Factor-1 (HIF-1) signaling pathways [10,11]. Both transcription factors are involved in key processes regarding cancer such as inflammation, cell proliferation and survival, apoptosis, and angiogenesis [8,12,13].

To date, the ability to inhibit the NF-kB pathway has not been studied comparatively for the CIGB-552 and its parental peptide LALF_32–51_. We aimed to assess if this property was selected during the screening of the peptide library, or it was partly driven by LALF_32–51_ immunomodulatory properties [3,6,14]. On the other hand, to further investigate the role of COMMD1 in CIGB-552 mechanism of action we generated a COMMD1 knock-out (KO) cell line and performed a cDNA array to compare both, KO and wild type (WT) cell lines. Finally, the contribution of NF-kB and HIF-1 transcriptional regulation upon CIGB-552 treatment in KO and WT cells was evaluated.

## 2. Results

### 2.1. CIGB-552 but Not LALF_32–51_ Mediates NF-kB Activity in HT-29 and H460 Reporter Cell Lines

The modulation of the inflammatory response has been reported for both CIGB-552 [14] and LALF_32–51_ [2,3] peptides. However, it is not well established how this effect may have a direct impact on anti-cancer properties. Since CIGB-552 cytotoxicity has been characterized in several cancer cell lines [1,4,5], we first decided to determine the cytotoxicity of LALF_32–51_ for the parental lung and colon cancer cell lines used in these assays. As shown in Table 1, CIGB-552 has the most cytotoxic effect on both studied cell lines and the same cell-line dependent sensitivity was observed for CIGB-552 and LALF_32–51_. To better understand the selection of CIGB-552 as a new anticancer peptide, the effect on NF-κB mediated transcription of both peptides was evaluated in two reporter cell lines derived from H460 and HT-29 cancer cells [15,16]. The cells were seeded in 96-well plates and cultured for 24 h before adding the peptides and the pro-inflammatory stimulus: Tumor necrosis factor alpha (TNF-α). The chosen peptide concentrations used in these assays correspond to the Inhibitory Concentration 50 (IC_50_) and half the IC_50_ of the CIGB-552 for each cell line, and for the stimulus, the effective concentration 50 (EC_50_) described for each reporter cell line [15,16].

**Figure 1:**
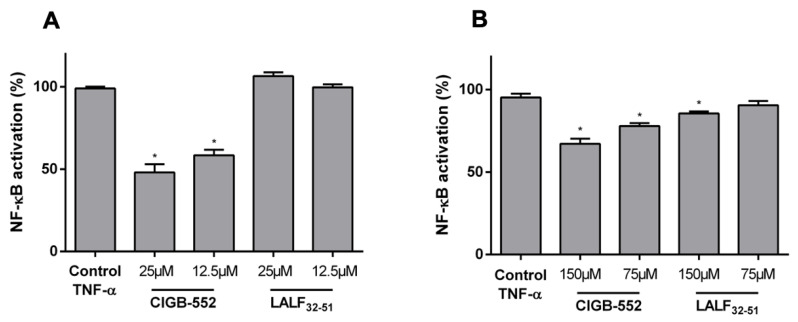
Evaluation of TNF-α-induced NF-κB activation in reporter cell lines treated with CIGB-552 or LALF_32–51_. H460-NF-κB-hrGFP (**A**) and HT-29-NF-κB-hrGFP (**B**) were treated with the peptides and TNF-α for 24 and 48 h, respectively, and GFP expression was assessed by flow cytometry considering 10,000 single cells for each sample. NF-κB activation was calculated considering GFP expression of the TNF-α-stimulated control as 100% activation and data were normalized to this 100% control. Data represented as the mean ± SD of technical triplicates of one representative experiment out of three. One-way ANOVA analysis (Dunnett’s post-test) was applied to compare the treated groups with the control condition, * *p* < 0.05.

As shown in Figure 1, CIGB-552 was capable of inhibiting the NF-κB response induced by TNF-α in both cell lines. Furthermore, the extent of this modulation in each cell line confirms the higher sensitivity of the H460 cell line than HT-29 cells to CIGB-552 treatment. On the other hand, LALF_32–51_ only showed a slight effect on NF-κB activation in HT-29-NF-κB-hrGFP cell line, but no activity on H460-NF-κB-hrGFP, showing that at the same concentration used for CIGB-552, the parental peptide is less efficient in modulating NF-κB signaling pathway.

Since both peptides have cell-penetrating properties, and none of them was previously studied in more complex in vitro systems, the same reporter assay was performed in a 3D culture system by using spheroids. As opposed to cell monolayers which are 2D cultures, 3D cell cultures are well documented to better mimic the in vivo situation, regaining intrinsic cellular properties. Regarding tumor biology, spheroids resemble the proliferating, quiescent, and dying cells that coexist in normoxic, hypoxic, or necrotic zones within the 3D arrangement [17].

**Figure 2:**
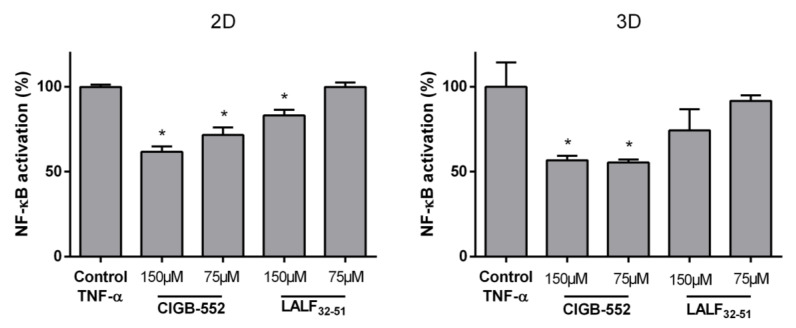
Evaluation of TNF-α-induced NF-κB transcription HT-29-NF-κB-hrGFP spheroids. Cells were seeded on non-adherent (3D) or adherent (2D) surfaces and cultured for four days. Cells were treated with the peptides and TNF-α for 24 h. GFP expression was assessed by flow cytometry considering 10,000 single cells for each sample. NF-κB activation was calculated considering GFP expression of the TNF-α-stimulated control as 100% activation and data were normalized to this 100% control. Data represented as the mean ± SD of technical triplicates of one representative experiment out of three. One-way ANOVA analysis (Dunnett’s post-test) were applied to compare the treated groups with the control, * *p* < 0.05.

Using spheroid culture we aimed to determine if both peptides retained their capacity of modulating NF-κB signaling pathway in HT-29-NF-κB-hrGFP (Figure 2). In the 3D model of spheroids, only CIGB-552 was able to inhibit the NF-κB activity induced by TNF-α and non-significant differences were detected between the obtained modulation using 2D and 3D cultures (Figure 2). These results highlight the precise and adequate selection of CIGB-552 as a novel anticancer cell penetrating peptide.

### 2.2. Interaction between CIGB-552 and COMMD1 by In Situ Immunodetection

Interaction between CIGB-552 and its molecular target COMMD1 has been previously reported by pull-down [4] and competitive enzyme-linked immunosorbent assay [18]. In addition, a protein complementation assay in vitro was reported [5]. However, since CIGB-552 is a synthetic peptide with modifications that cannot be replicated in vitro by the cells, the complementation was performed with the L2 peptide, which represents the primary sequence that has been modified in order to generate the CIGB-552 peptide, without D-amino acids and without N-terminal acylation (see Table 1). COMMD1 is mainly located in the cytoplasm but has the capacity to translocate to the nucleus where it acts as a negative regulator of NF-κB mediated transcription. According to internalization and localization studies, CIGB-552 also can be found both in the cytoplasm and nucleus [5]. Therefore, we evaluated if co-localization of COMMD1 and CIGB-552 was detectable in H460 and HT-29 cell lines.

**Figure 3:**
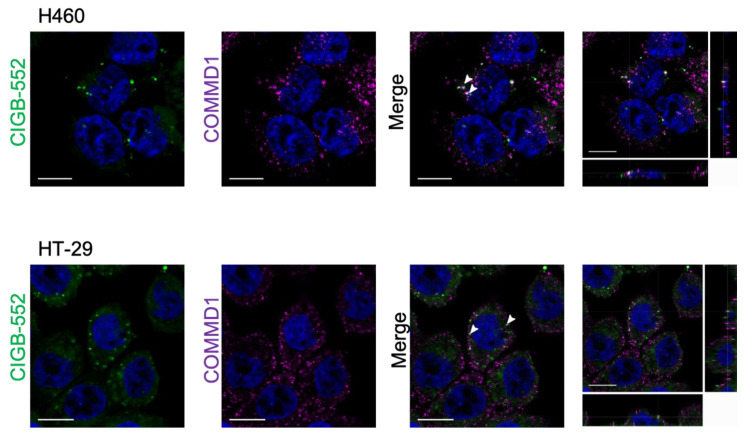
Interaction between CIGB-552 and COMMD1 in H460 and HT-29. Cells were incubated with 100 μM of CIGB-552 conjugated to FITC (green) and COMMD1 in situ interaction was detected 24 h later by confocal microscopy. Nuclei were labelled using TOPRO-3 probe (blue) and COMMD1 was detected with a secondary antibody labelled with Cy5 (magenta) (scale bar = 10 µm).

Results obtained by in situ immunodetection of COMMD1 after internalization of fluorescein isothiocyanate (FITC)-conjugated peptide showed few clusters of CIGB-552 in the vicinity where COMMD1 was located (Figure 3). These results do not allow quantifying the colocalization of both molecules. However, the punctuated pattern observed in internalized CIGB-552 and endogenous COMMD1 suggest a probable interaction.

### 2.3. CIGB-552 Treatment in H460 WT and COMMD1 KO Cell Lines

**Figure 4:**
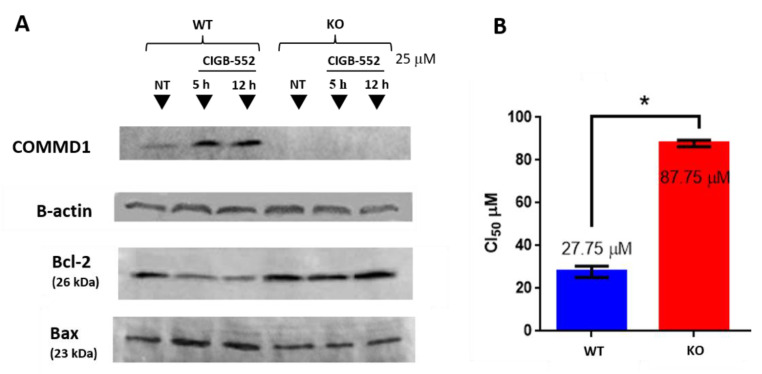
Generation of the H460 COMMD1 KO cell line. (**A**) H460 WT and KO cell lines treated with CIGB-552 (25 µM) for indicated times and COMMD1, Bcl-2 and Bax were determined by Western blot analysis. Actin was used as a control for protein loading (**B**) CIGB-552 was added to 10,000 cells (0–200 μM) and incubated for 48 h. Cell viability was determined by sulforhodamine B sodium salt assay and the IC50 values were calculated. Data presented as the mean ± SD. * *p* < 0.05.

To further investigate the relevance of COMMD1 in the mechanism of action of CIGB-552, we generated a COMMD1 knock-out cell line. H460 was selected to perform the knock-out of COMMD1 by CRISPR-Cas9 because it was the most sensitive cell line to the CIGB-552 effect. H460 cell line was transfected with a plasmid containing the Cas9 and the COMMD1 guide and another plasmid with a functional cassette containing GFP, puromycin resistance and homology arms for homologous recombination (see Supplementary Figure S1). Absence of COMMD1 in the generated clones was evaluated by sequencing (Supplementary Figure S1B) and Western blot analysis (Figure 4A). Next, we examined the levels of proapoptotic protein Bax and antiapoptotic protein Bcl-2 in cytosolic extracts of WT and KO cells. As shown in Figure 4A, the protein Bax was markedly induced, whereas Bcl-2 was significantly inhibited after treatment with the peptide in WT cells. This suggests that the apoptotic effect of CIGB-552 is partly caused by upregulating the Bax/Bcl-2 protein ratio, which is a critical determinant of apoptosis. This effect was abolished in KO cells indicating that COMMD1 has a functional role in the apoptotic activity of CIGB-552. These results are in concordance with the previously reported in H460 knockdown cell line, where after silencing COMMD1, CIGB-552-induced apoptosis was impaired [4]. When comparing the cytotoxic effect of CIGB-552 in both cell lines, KO cells presented higher IC_50_ value than WT cells (Figure 4B). However, since the cytotoxic activity of the peptide was not completely impaired in knock-out cells, we do not rule out that other factors may be involved in the cytotoxic activity of the peptide.

**Figure 5:**
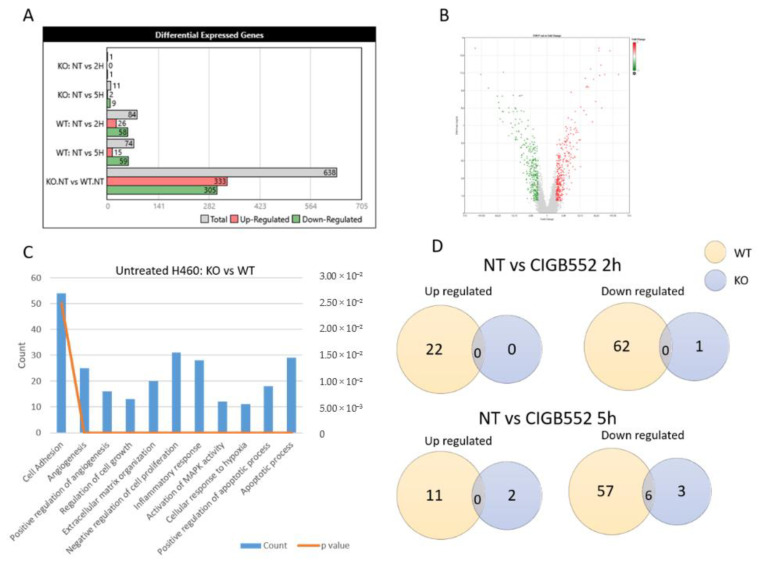
DNA microarray in H460 WT and KO cells. Cells were treated with 25 µM or without CIGB-552 for 2 and 5 h. (**A**) Differential expressed genes (DEGs) among different conditions. (**B**) Volcano plot of DEGs in WT and KO cell line without treatment. (**C**) Functional enrichment of biological processes of DEGs in H460 WT and KO. (**D**) Venn diagram for comparison of DEGs up- and downregulated in KO and WT cells without treatment (NT) and treated with CIGB-552.

Once the KO cell line was obtained, a DNA microarray was performed using both KO and WT cell lines treated with the peptide. The most contrast in the number of differentially expressed genes was observed in the untreated conditions (Figure 5A,B). Nevertheless, in all cases, the study of biological processes that differ in the KO and WT samples shows pathways that are essential for carcinogenesis such as: regulation of cell growth, angiogenesis and apoptosis, response to hypoxia, and inflammatory response (Figure 5C). The few genes with differential expression in the KO cell line were detected in cells treated with CIGB-552 for 5 h but only six of these genes were also differentially expressed in the WT cell line (Figure 5D). All DEGs lists of array analysis are available in Supplementary Table S1.

When comparing the effects of the KO of COMMD1 (Table 2), there are some NF-kB targets upregulated in the KO cell line, such as IL1A, IL1B, CXCL1, CXCL5, and IL6R, indicating that COMMD1 may be negatively regulating NF-kB activation. However, there are also other targets that act inhibiting NF-kB, such as inhibitors NFKBIA and NFKBID, which were upregulated in the KO cell line. With regard to HIF-1, many related genes were upregulated in the absence of COMMD1, for example GATA6, STC1, C5AR1, and PTGS2, as well as other genes involved in angiogenesis, such as ANPEP (CD13). On the other hand, some genes related to HIF-1 activity were downregulated in the KO such as VEGFC and CTGF.

Even though CIGB-552 had little effect on the KO cell line (Table 3), some genes were upregulated by the peptide such as CYR61 and CTGF. Both genes were reported to inhibit proliferation and led to growth arrest in the H460 cell line [19,20]. There were also two genes that were altered by CIGB-552 treatment on both KO and WT cell lines. The induction of EGR1 has been reported to be induced by different drugs and to promote apoptosis in H460 cells [21,22,23]. Also, ID1 was downregulated by CIGB-552. ID1 protein associates with different transcription factors to regulate cell fate determination, differentiation, and induce angiogenesis [24,25]. These results show that CIGB-552 has also apoptotic and anti-angiogenic effects that are independent of COMMD1. Conversely, some inflammation related genes were also modified in WT cells upon treatment with CIGB-552 (Table 3). For instance, MAP3k14, ZFP36, and NFKBIA are genes related to the NF-kB signaling pathway.

### 2.4. CIGB-552 Requires COMMD1 to Exert Its Effect on Inflammation and Angiogenesis in the H460 Cell Line

COMMD1, a major interactor of CIGB-552, is a pleiotropic protein, and it was found to have a role in many signaling pathways implicated in carcinogenesis including NF-κB and HIF-1 [11,26]. In order to evaluate the relevance of COMMD1 as a novel target for the anticancer properties of CIGB-552, the effect of the CIGB-552 in NF-κB and HIF-1 signaling pathways was analyzed using different luciferase-based reporter assays and comparing the WT and KO cell lines.

#### 2.4.1. CIGB-552 Effects on NF-κB Transcriptional Activity Are Impaired in H460 COMMD1 KO Cells

As previously shown, part of the anti-tumor effects of CIGB-552 are driven by NF-κB inhibition. For assessing the importance of COMMD1 in this process, the H460 WT and the COMMD1 KO cell lines were transiently transfected with a reporter plasmid containing NF-κB response elements upstream of the firefly luciferase gene. The cells were then treated with different concentrations of CIGB-552 peptide and TNF-α as an inductor of NF-κB activation. Cells were lysed after 24 h and luciferase activity was measured.

**Figure 6:**
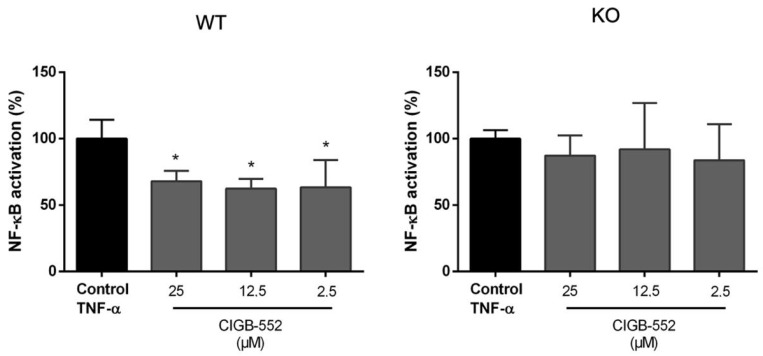
Evaluation of TNF-α-induced NF-κB activation in H460 WT and COMMD1 KO cell lines. Cells were transfected with a pNFκB-luc plasmid and after 24 h were treated with 40 ng/mL TNF-α and different concentrations of CIGB-552. After 24 h, luciferase activity was assessed, normalized by protein content. NF-kB activation was calculated considering the TNF-α-stimulated control as 100% activation and data were normalized to this control. Data are represented as the mean ± SD of technical triplicates of one representative experiment out of three. One-way ANOVA analysis (Dunnett’s post-test) was applied to compare the treated groups with the TNF-α control, * *p* < 0.05.

In the KO cell line, no modulation of the NF-κB activation was found at the concentrations of the peptide evaluated (Figure 6). While in the presence of COMMD1, CIGB-552 was capable of reducing the NF-κB activation at all concentrations tested.

#### 2.4.2. CIGB-552 Effects on HIF-1 Transcriptional Activity Are Impaired in H460 COMMD1 KO Cells

**Figure 7:**
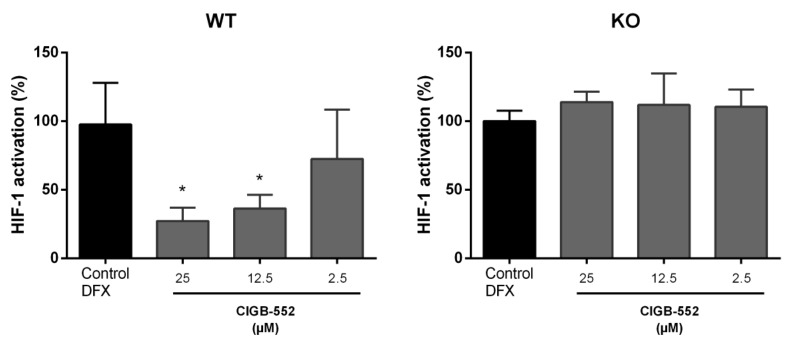
CIGB-552 modulates DFX-induced HIF-1 activation only in H460 WT cells. H460 WT and COMMD1 KO cells were transfected with a pHRE-EPO-luc plasmid and treated with different concentrations of CIGB-552 and 150 μM DFX for 24 h. Luciferase activity was assessed on cell lysates, normalized by protein content. HIF-1 activation was calculated considering the DFX-stimulated control as 100% activation and data were normalized to this control. Data are represented as the mean ± SD of technical triplicates of one representative experiment out of three. One-way ANOVA analysis (Dunnett’s post-test) was applied to compare the treated groups with the DFX control, * *p* < 0.05.

Besides to contributing to the termination of the NF-κB response, COMMD1 is implicated in the hypoxia induced factor HIF-1 signaling pathway [27]. HIF-1 is also a transcription factor regarded as a potential target for cancer therapies [12,28]. Genes induced by HIF-1 activation include, among others, genes related to angiogenesis, cell proliferation, glucose metabolism, and extracellular matrix remodeling [12]. It was previously reported that CIGB-552 presented some anti-angiogenic properties in a xenograft mice model [7], but it was not studied if the HIF-1 pathway was involved in this effect. Since regulation of angiogenesis and response to hypoxia were processes identified in the DNA microarray analysis, it seemed interesting to study whether CIGB-552 may have a direct effect on HIF-1 activation. By using a reporter plasmid to evaluate HIF-1 activation (pHRE-EPO-luc) we aimed to assess the CIGB-552 effect on HIF-1 pathway on both WT and COMMD1 KO cell lines. Cells were transiently transfected with the reporter plasmid and treated with the peptide 4 h later. Finally, a hypoxia-mimetic agent (deferoxamine, DFX) was added to stimulate the HIF-1 mediated response. DFX was able to induce the same levels of HIF-1 transcriptional activity on WT and KO cells (data not shown). As indicate in Figure 7, only in the presence of COMMD1, CIGB-552 managed to reduce hypoxia-induced HIF-1 activation in a dose-dependent manner.

## 3. Discussion

Cancer remains a prevalent disease and one of the leading causes of death worldwide [29]. In spite of recent advances in diagnosis and treatment, there is still a need for new drugs with less cytotoxicity and treatment resistance. Therapeutic peptides have attracted attention for cancer treatment. Their therapeutic potential lies in its ability to display high selectivity and lack of adverse toxic effects among other advantages [30]. CIGB-552 is a synthetic peptide with anticancer properties, selective cytotoxicity towards tumoral cell lines and responsible for increasing survival in tumor-bearing mice [1,4,7]. Its anti-tumor activity is, at least in part, mediated by the stabilization of the COMMD1 protein. This protein is a transcriptional regulator that acts negatively, regulating NF-κB and HIF-1 signaling pathways [11]. COMMD1 is predominantly localized in the cytoplasm and to a lesser extent in the nucleus, where it appears to play an important role in preventing NF-κB and HIF-1-mediated transcription [11]. Given the role that these two transcription factors have in tumor progression and response to treatment, the possibility to modulate their activity through new drug candidates has drawn attention [31].

Early studies showed that CIGB-552 induces ubiquitination and proteasomal degradation of RelA, a NF-κB subunit, in H460 cells [4]. Additionally, CIGB-552 treatment negatively regulated NF-κB activation and IL-8 production in HT-29-NF-κB-hrGFP cells [14]. On the other hand, the parental peptide LALF_32–51_ is a cell-penetrating peptide that has shown to modulate LPS-induced cytokine gene expression in vivo [3]. However, up to the present report it was still unknown if the mechanisms of these LALF_32–51_ effects may be related to NF-κB activity or it is a gained characteristic from the selection of CIGB-552 based on the Ala-library screening. An example of the CIGB-552 election as an anticancer peptide is the IC_50_ value of LALF_32–51_ that was higher than for CIGB-552 in both cell lines analyzed. Therefore, a reporter assay for comparing the effect on NF-κB of CIGB-552 and its parental peptide LALF_32–51_ was performed. Our results showed that CIGB-552 was able to modulate the NF-κB transcriptional activity induced by TNF-α in both cell lines. It should be noted that the extent to which CIGB-552 diminished NF-κB activation in both cell lines was different and in accordance with previous studies indicating that H460 had higher COMMD1 expression and higher peptide internalization compared to HT-29 cell line [5]. LALF_32–51_ had only a slight effect on HT-29 cells but only at the highest concentration tested. To improve the culture system on which the assay was performed, 3D cell culture of the reporter model were incorporated. Recently it was reported that HT-29 spheroids showed considerably strong changes in cell proliferation and metabolic capacity when compared to those obtained in 2D cultures [17]. Taking into account these findings, we wanted to extent our 2D results from HT-29 reporter cell lines to spheroids models. 3D cell cultures are well documented to preserve intrinsic characteristics and to better mimic the in vivo situation than monolayers of cells cultured on plastic [32]. For instance, proliferating, quiescent and dead cells coexist in all the oxygen gradient zones within spheroids just as in solid tumors [33]. Moreover, gene expression profiles and responses to treatment have been better represented in 3D spheroid models [17]. Consequently, the potential of 3D cancer models as more biologically relevant for anticancer drug development is increasingly recognized. In HT-29-NF-κB-hrGFP spheroids, CIGB-552 preserved its ability to inhibit the NF-κB transcriptional activity whereas LALF_32–51_ had no effect on this 3D model, validating CIGB-552 as a better therapeutic candidate for NF-κB inhibition. Overall, our results indicate that CIGB-552 outperforms its parental peptide LALF_32–51_ in its cytotoxic and anti-inflammatory activity on H460 and HT-29 cell lines. In addition, we decided to investigate the in situ interaction between CIGB-552 and COMMD1 in H460 and HT-29 cells. It was reported that there are differences in COMMD1 expression and CIGB-552 internalization capacity between cell lines, being H460 the one with the greatest COMMD1 expression and more susceptible to CIGB-552 treatment [5]. Additionally, we had previously aimed to study this interaction with a protein complementation assay (PCA), but since CIGB-552 is a synthetic peptide that possess modified amino acids (D amino acids) it cannot be translated inside cells. Thus, the interaction was detected by using the L2 peptide, which has the same primary structure but without modifications on transfected cells. For that reason we decided to study the interaction with endogenous COMMD1 after FITC-conjugated peptide internalization. While the effect was not clear and elegant enough to get a quantitative measure of co-localization, we showed accumulation of CIGB-552 near foci where endogenous COMMD1 was located. Although in situ immunodetection showed some limitations, it is a more relevant sign of interaction since none of the molecules was overexpressed and no transfection was required, processes that may stress the cell physiology and machinery.

As mentioned, CIGB-552 interacts with COMMD1 protein to exert its cytotoxic effect. It was previously reported that silencing COMMD1 affected CIGB-552 pro-apoptotic activity [4]. On the other hand, a proteomic approach performed on HT-29 cells treated with the peptide revealed that major processes affected by CIGB-552 treatment were apoptosis, oxidative damage, inflammation response, cell adhesion, and motility [14]. With the aim of elucidating the role of COMMD1 in the CIGB-552 anticancer properties, the effect of CIGB-552 in WT and COMMD1 KO H460 cell lines was evaluated with a DNA microarray. A significant number of DEGs (see Supplementary Table S1) of cells treated with the peptide compared to non-treated controls were detected mainly in WT cells. These genes were involved in key processes implicated in cancer, such as inflammation and cell death. In the KO cell line, CIGB-552 treatment had little effect on gene expression. Probably using 25 μM of peptide is not enough to induce some effect in KO cells at 2 and 5 h. Additionally, the KO of COMMD1 does not account for all the genes that are affected by CIGB-552. However, the minimal effect that the peptide had on COMMD1 KO gene expression may support the relevant role of COMMD1 in the anti-tumor activity triggered by CIGB-552. However, when comparing only non-treated cells there was a significant number of DEGs in KO and wild-type cells. GO enrichment analysis showed that among these DEGs were genes involved in regulation of cell proliferation, apoptosis and angiogenesis, extracellular matrix organization and response to hypoxia and inflammation. These results highlight the importance of COMMD1 in CIGB-552 response in H460 cells. On the other hand, we found only two genes that are modulated by CIGB-552 treatment in both KO and WT cells, suggesting that COMMD1 is dispensable for part of the effects elicited on H460 cells. Conversely, key genes involved in the NF-kB pathway and angiogenesis process were modulated only in WT treated cells. It was previously reported that CIGB-552 has an impact on cell redox capacity of H460 cells by altering SOD-1 activity, a protein regarded as a major nuclear superoxide scavenger [4]. The maintenance of cell redox balance is ensured by many antioxidant systems whereas a large number of stress-inducible transcription factors are redox-sensitive such as NF-κB, HIF1α, and p53 [34]. Furthermore, CIGB-552 induced regulation of a series of proteins leading to proteasomal degradation of HIF1α in HT-29 cells [14]. Apart from being identified as an inhibitor of NF-κB activity, there is evidence for a role of COMMD1 in HIF-1 signaling [11]. COMMD1 inhibits HIF-1 activity by interacting with HIF-1α, in a similar way to the inhibitory effects of COMMD1 on NF-κB activity [8]. As NF-κB, HIF-1 is a dimeric transcription factor that can shuttle between the nucleus and the cytoplasm but regulates the expression of genes involved in energy metabolism, cell growth and angiogenesis [12]. Based on these findings, and the fact that some NF-kB and HIF1 related genes were affected on the KO cell line, we performed the NF-κB and HIF-1 modulation assay in WT and KO H460 cell lines. Only WT H460 cells treated with CIGB-552 diminished the induced activation of NF-κB and HIF-1, which remarks the importance of COMMD1 as a molecular target for CIGB-552. For the first time we report the activity of CIGB-552 on HIF-1-mediated transcription. Given the role of this transcription factor in angiogenesis, this result may explain the anti-angiogenic effect of CIGB-552 reported on a HT-29 xenograft mouse model [7]. Moreover, these results show that COMMD1 is necessary for HIF-1 and NF-κB modulation induced by CIGB-552. It should be noted that while the DEGs observed on the array analysis correspond to the effect of CIGB-552 on KO and WT cell lines, the peptide effects detected on the reporter assays regarding NF-kB and HIF-1 activity refer to stimulated (TNF-α or DFX) conditions. In both cases, COMMD1 demonstrated to play a key role in CIGB-552 anti-inflammatory and anti-angiogenic effects.

Altogether, our data indicate that CIGB-552 has been successfully selected as an anti-tumor peptide as a result of the screening process from LALF_32–51_. The presence of the COMMD1 protein is crucial for the effects evaluated in intestinal and lung tumor cell lines including HIF-1 and NF-κB modulation activity.

## 4. Materials and Methods

### 4.1. Reagents and Chemicals

Unless otherwise indicated, all chemicals used were of the highest grade available and were purchased from Sigma-Aldrich (St. Louis, MO, USA). Culture media, fetal bovine serum (FBS), and consumables for cell culture were obtained from Life Technologies (Carlsbad, CA, USA), GE Healthcare, and Greiner. All reagents for peptide synthesis were of synthesis grade. Reagents for chromatography were of high-performance liquid chromatography (HPLC) grade.

### 4.2. Peptide Synthesis

Peptide CIGB-552 and LALF_32–51_ were synthesized on a solid phase and purified by reverse-phase-HPLC to >95% purity on an acetonitrile/H_2_O trifluoroacetic acid gradient and confirmed by ion-spray mass spectrometry (Micromass, Manchester, UK). Lyophilized peptides were reconstituted in PBS for in vitro experiments. The carboxyfluorescein fluorophore was attached selectively by an amide bond to the N-terminus of the peptide sequences during the synthesis of the peptide in solid phase performed using the Fmoc/t-Bu chemistry. The linking is direct to the N-terminus of the peptide; there are no additional residues.

### 4.3. Cell Culture

H460 (ATCC, HTB-177), HT-29 (ATCC, HTB-38), H460-NF-κB-hrGFP 2G6, and HT-29-NF-κB-hrGFP E5 cells [11] were cultured in RPMI 1640 containing Glutamax and supplemented with 10% (*v*/*v*) FBS. Cells were routinely propagated in 25 or 75 cm^2^ tissue culture flasks at 37 °C, 5% CO_2_ in a humidified incubator until reaching approximately 70% confluence. Cells were subsequently trypsinized, the concentration was adjusted, and used for different experimental settings. Cells were cultured for no longer than 10–15 passages.

### 4.4. NF-κB Activation Studies Using Stable Reporter Cell Lines

Cells were seeded in 96-well plates in RPMI 1640 supplemented with 10% (*v*/*v*) FBS with a seeding density of 3.0 × 10^4^ cells/well and cultured overnight. Cells were then subjected to different concentrations of peptide CIGB-552 or LALF_32–51_ and TNF-α. Cells were further incubated for 24 h for HT-29-NF-κB-hrGFP and 48 h for H460-NF-κB-hrGFP and subsequently trypsinized and analyzed by flow cytometry. GFP expression and viability using propidium iodide (PI) were determined using a BD Accuri^TM^ C6 (BD Biosciences, San Jose, CA, USA) flow cytometer equipped with 488 and 633 nm lasers. BD Accuri^TM^ C6 software was used for data acquisition. GFP and PI fluorescence emission was detected using band-pass filters 533/30 and 585/40, respectively. For each sample, 10,000 single events gated on a forward scatter (FSC) versus side scatter (SSC) dot plot were recorded. Only single living cells (those that excluded PI) were considered for results comparison (see Supplementary Figure S2). In order to consider valid each assay, the viability of all samples (according to PI staining) must be over 95 percent. Data were analyzed using FlowJo vX.0.7 (Tree Star Inc, Ashland, OR, USA) software. Cells without treatment and cells treated only with the stimulus (TNF-α) or the different compounds were included as controls. NF-κB activation was calculated subtracting the value of % GFP+ cells of non-stimulated controls to each condition and was normalized considering the TNF-α control as 100% NF-κB activation.

### 4.5. HT-29-NF-κB-hrGFP Spheroid Generation and Culture

Agarose 1.5 % (*w*/*v*) was used to generate a non-adherent surface in a 96-multiwell plate by placing 50 μL per well and allowing the agarose to solidify at room temperature. HT-29-NF-κB-hrGFP cells (3 × 10^4^/well) were seeded for the spheroid culture (3D), and for the control in adherent 96-multiwell plate (2D), 1.5 × 10^4^ cells/well. Cells were cultured for four days before adding CIGB-552 or LALF_32–51_. At the same time TNF-α was added: 2.5 ng/mL and 5 ng/mL, for 2D and 3D cultures, respectively, considering the EC_50_ for each type of culture. Cells were further incubated 24 h and GFP expression was analyzed by flow cytometry. NF-κB activation was calculated as described above.

### 4.6. Confocal Microscopy

Cells were plated in 12-well plate (1 × 10^5^ cells/well) containing sterile glass cover slips and cultured for 24 h. FITC-conjugated CIGB-552 (100 μM) was added for one hour and then washed. Cells were fixed in 4% (*w*/*v*) paraformaldehyde (PFA) for 15 min, permeabilized using 0.2% (*v*/*v*) Tween 20 solution in PBS (Tween-PBS). For COMMD1 immunodetection, permeabilized cells were blocked with 2% (*w*/*v*) bovine serum albumin in PBS (BSA-PBS) for 1 h. Primary antibody, mouse monoclonal anti-COMMD1 (M01 clone 2A12, Abnova, Taipei, Taiwan) was diluted 1:500 in BSA-PBS and incubated overnight at 4 °C. Three washing steps were performed using 0.2% (*v*/*v*) Tween 20 solution in PBS solution. Goat anti-Mouse IgG (H + L) secondary antibody, Cy3 conjugated (Life Technologies, ThermoFisher Scientific, Waltham, MA, USA) diluted 1:1000 in Tween-PBS was added and incubated for 1:30 h. Finally, nuclei were stained with TOPRO-3 diluted 1:1000 (Life Technologies, ThermoFisher Scientific, Waltham, MA, USA) and incubated for 30 min. All images were taken using laser confocal microscope Leica TCS SP5 equipped with a 63X NA 1.42 oil immersion objective (Leica Microsystems, Wetzlar, Germany). In order to assess fluorescence intensity both in cytoplasm and nuclei, images were taken in identical conditions (laser power, photomultiplier voltage, line and frame average, and zoom) for all cell lines and 10 single optical sections were used for the analysis.

### 4.7. COMMD1 Knock-out in H460 Cells by CRISPR-Cas9

The CRISPR COMMD1 knock-out Kit was purchased from OriGene (Rockville, MD, USA), containing two pCas9 plasmids (targeted sequence of gRNA1 CGCATTCAGCAGCCCGCTCA and gRNA2 CGGGTACCCCGGCATCACAG, respectively), one pCas9 plasmid with a scrambled sequence as control and a repair donor DNA plasmid containing left and right homologous arms of COMMD1 and GFP-puro functional cassette. H460 cells were co-transfected with Turbofectin (OriGene, Rockville, MD, USA) using 1 μg of the repair donor DNA together with 1 μg of one of the three pCas9-Guide vectors. Seventy-two hours after the transfection medium was replaced with 1 μg/mL puromycin containing medium for selection of stably transfected cells. Array dilution method was used after six days of selection to obtain single cell clones for genotypic and phenotypic characterization [35]. At least 20 clones from two independent transfections experiments were analyzed. For genotyping analysis, DNA from individual clones was obtained using the Mini Kits QIAamp from QIAGEN (Germantown, MD, USA). Promega’s master mix was used (Madison, WI, USA) for the PCR reactions. DNA sequencing of selected clones was performed by Sanger using primers CF (TAAGCTGCCAACTCTGACCCC) and CR (AAGCCTTTCGGCTTGTGAGGA). From the clones that were confirmed to be KO, two were selected for phenotypic characterization by western blot as previously described [1].

### 4.8. HIF-1 and NF-kB Activation Studies in WT and COMMD1-KO H460 Cells

Cell lines NCI-H460 parental (WT) and NCI-H460 Kock out for COMMD1 (KO) were seeded in 24-multiwell plate and cultured for 24 h. Transient transfection was performed with pNF-kB-luc or pEPO-luc plasmids using Lipofectamine2000 (Life Technologies, ThermoFisher Scientific, Waltham, MA, USA) according to manufacturer instructions. At 5 h post-transfection and for HIF-1 transcriptional activity, cells were pre-incubated with CIGB-552 prior to the addition of the hypoxia-mimetic agent (Deferoxamine, 150 μM), while for cells transfected with pNF-kB-luc, the peptides and the pro-inflammatory stimulus (TNF-α, 30 ng/mL) were added simultaneously. After 24 h cells were lysed and luciferase activity was measured in a luminometer (Lumistar Optima, BMG Labtech, Ortenberg, Germany) and normalized according to total protein content determined by the bicinchoninic acid method using BCA Protein Assay Kit Sigma-Aldrich (St. Louis, MO, USA).

### 4.9. DNA Microarray

NCI-H460 and NCI-H460-B7 (knock-out for COMMD1 gene) cell lines were grown at a concentration of 2 × 10^5^ cells per well in 6-well plates and treated with 25 μM of CIGB-552 for 2 and 5 h or were left untreated. For each experimental time three biological replicas were obtained. The samples were subjected to total RNA preparation using RNeasy Qiagen kit, (Qiagen Sciences Inc, Germantown, MD, USA) and expression profiling was performed using the Affymetrix Human Clariom S Assay (Affymetrix, ThermoFisher Scientific, Waltham, MA, USA), which interrogates over 20,000 well-annotated genes. Data analysis of differentially expressed genes was performed on Affymetrix TAC software (V4 Affymetrix, ThermoFisher Scientific, Waltham, MA, USA). Gene expression data were log-transformed and changes were considered significant when FDR *p*-value was less than 0.1 at absolute fold changes greater than 2. For functional enrichment analysis Gprofiler and Gorilla tools were used.

### 4.10. Statistical Analysis

Data was expressed as the mean ± standard deviation (SD) of triplicates of one representative experiment. At least three independent experiments were performed. GraphPad Prism Software version 6 (San Diego, CA, USA) was used for statistic calculations. Differences were considered statistically significant when *p* < 0.05 using a one-way ANOVA test with Dunnett’s post-test.

## Figures and Tables

**Table 1:** CIGB-552 and analogs peptides biological properties and cytotoxicity.

Peptide	Sequence	Properties	H460 ^1^ (μM)	HT-29 ^1^ (μM)	Ref.
LALF_32–51_	HYRIKPTFRRLKWKYKGKFW	AMP/IMP/CPP	460 ± 25	549 ± 25	[3,6]
L-2	HARIKPTFRRLKWKYKGKFW	ACP/CCP	57 ± 6 ^2^	ND ^3^	[1]
CIGB-552	Ac-HARIK*p*TFRR*l*KWKYKGKFW	ACP/CCP	23 ± 8 ^2^	166 ± 66 ^2^	[4,5]

AMP, antimicrobial peptide; IMP, immunomodulatory peptide; CPP, cell penetrating peptide; ACP, anticancer peptide. ^1^ D-amino acids are presented in small italic character. ^1^ Data expressed as IC_50_ ± SD. ^2^ Values previously published. ^3^ Not determined.

**Table 2:** Selected DEGs in untreated H460 COMMD1 KO vs. WT.

Gene Symbol	Description	Fold Change
*Angiogenesis*		
VEGFC	vascular endothelial growth factor C	−3.67
CTGF	connective tissue growth factor	−2.98
GATA6	GATA binding protein 6	4.64
STC1	stanniocalcin 1	2.72
PTGS2	prostaglandin-endoperoxide synthase 2 (cyclooxygenase 2)	9.22
ANPEP	alanyl aminopeptidase, membrane (ANP, CD13)	2.90
C5AR1	complement component 5a receptor 1	2.57
*Inflammation*		
IL1A	interleukin 1 alpha	2.33
IL1B	interleukin 1 beta	6.1
CXCL1	chemokine (C-X-C motif) ligand 1	2.30
CXCL5	chemokine (C-X-C motif) ligand 5	2.12
IL6R	interleukin 6 receptor	3.62
NFKBIA	nuclear factor of kappa light polypeptide gene enhancer in B-cells inhibitor, alpha	2.53
NFKBID	nuclear factor of kappa light polypeptide gene enhancer in B-cells inhibitor, delta	3.42

**Table 3:** Selected DEGs in untreated vs. CIGB-552-treated H460 cells.

Gene Symbol	Description	Fold Change
**COMMD1 KO**		
EGR1	early growth response 1	−90.15
ID1	inhibitor of DNA binding 1, dominant negative helix-loop-helix protein	2.87
CYR61	cysteine-rich, angiogenic inducer, 61	−2.32
CTGF	connective tissue growth factor	−2.22
**WT**		
EGR1	early growth response 1	−704.56
ID1	inhibitor of DNA binding 1, dominant negative helix-loop-helix protein	2.97
MAP3K14	mitogen-activated protein kinase kinase kinase 14	3.01
ZFP36	ZFP36 Ring Finger Protein (Tristetraprolin)	−6.33
NFKBIA	nuclear factor of kappa light polypeptide gene enhancer in B-cells inhibitor, alpha	−2.90
BCL2L11	BCL2-like 11 (apoptosis facilitator)	−2.14

## Data Availability

The data presented in this study are available on request from the corresponding author.

## Supplementary Materials

The following are available online, Figure S1. Generation of H460 COMMD1 KO cell line through CRISPR/Cas9. Table S1. DEGs obtained from microarray data. Figure S2. Gating strategy used for flow cytometry analysis of NF-kB activation.

## Sample Availability

Samples are available from the authors.
