# Supplementary material for: The Anticancer Peptide CIGB-552 Exerts Anti-Inflammatory and Anti-Angiogenic Effects through COMMD1
Source: Molecules. 2020 Dec 31;26(1):152. doi: 10.3390/molecules26010152 (PMC7795859; doi:10.3390/molecules26010152)
Supplement: Supplementary file 1 [file molecules-26-00152-s001.zip › Supplementary Figures_Daghero_et al.pptx]

## Slide 1
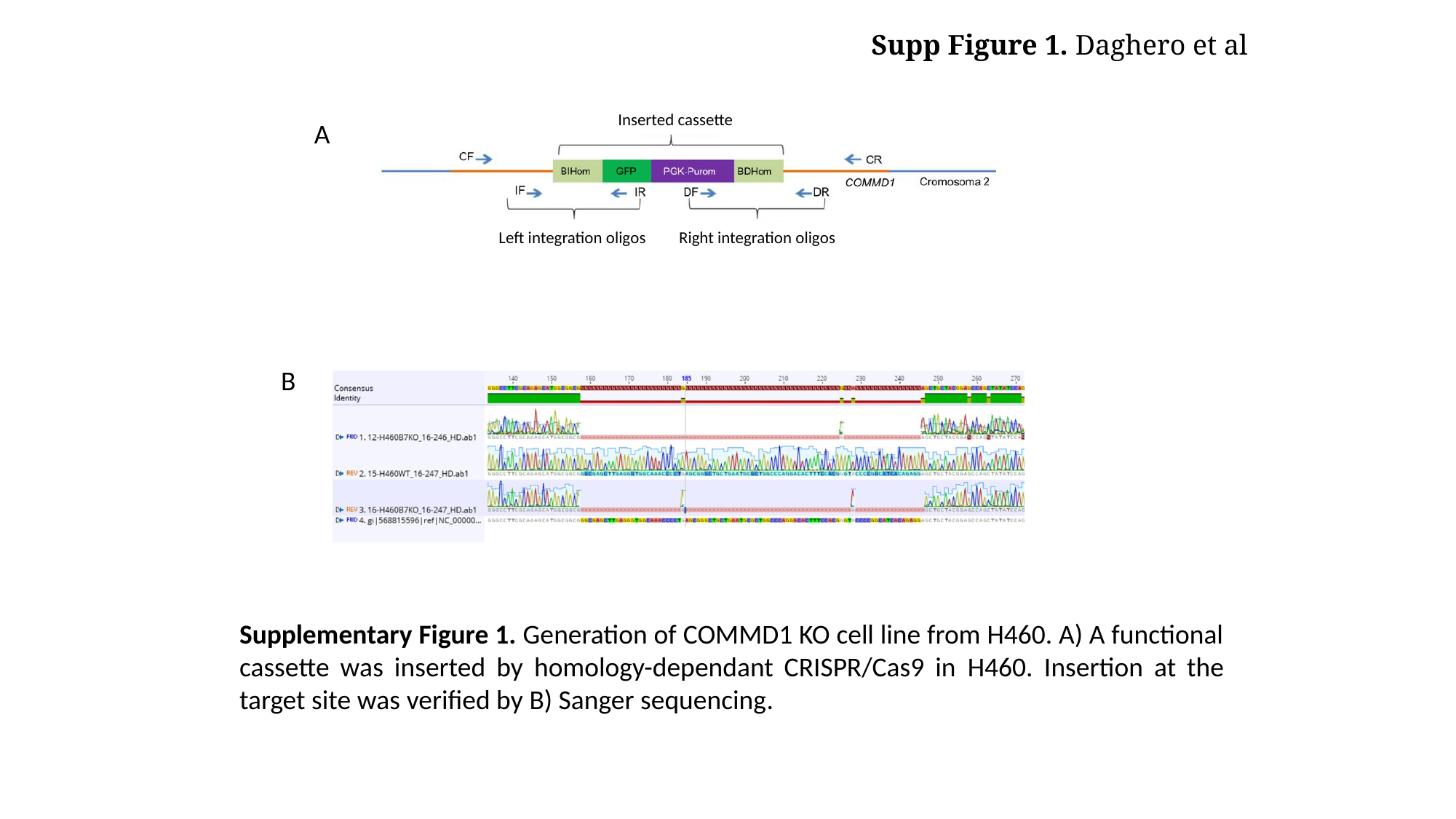

Supp Figure 1. Daghero et al
Inserted cassette
A
Left integration oligos
Right integration oligos
B
Supplementary Figure 1. Generation of COMMD1 KO cell line from H460. A) A functional cassette was inserted by homology-dependant CRISPR/Cas9 in H460. Insertion at the target site was verified by B) Sanger sequencing.

## Slide 2
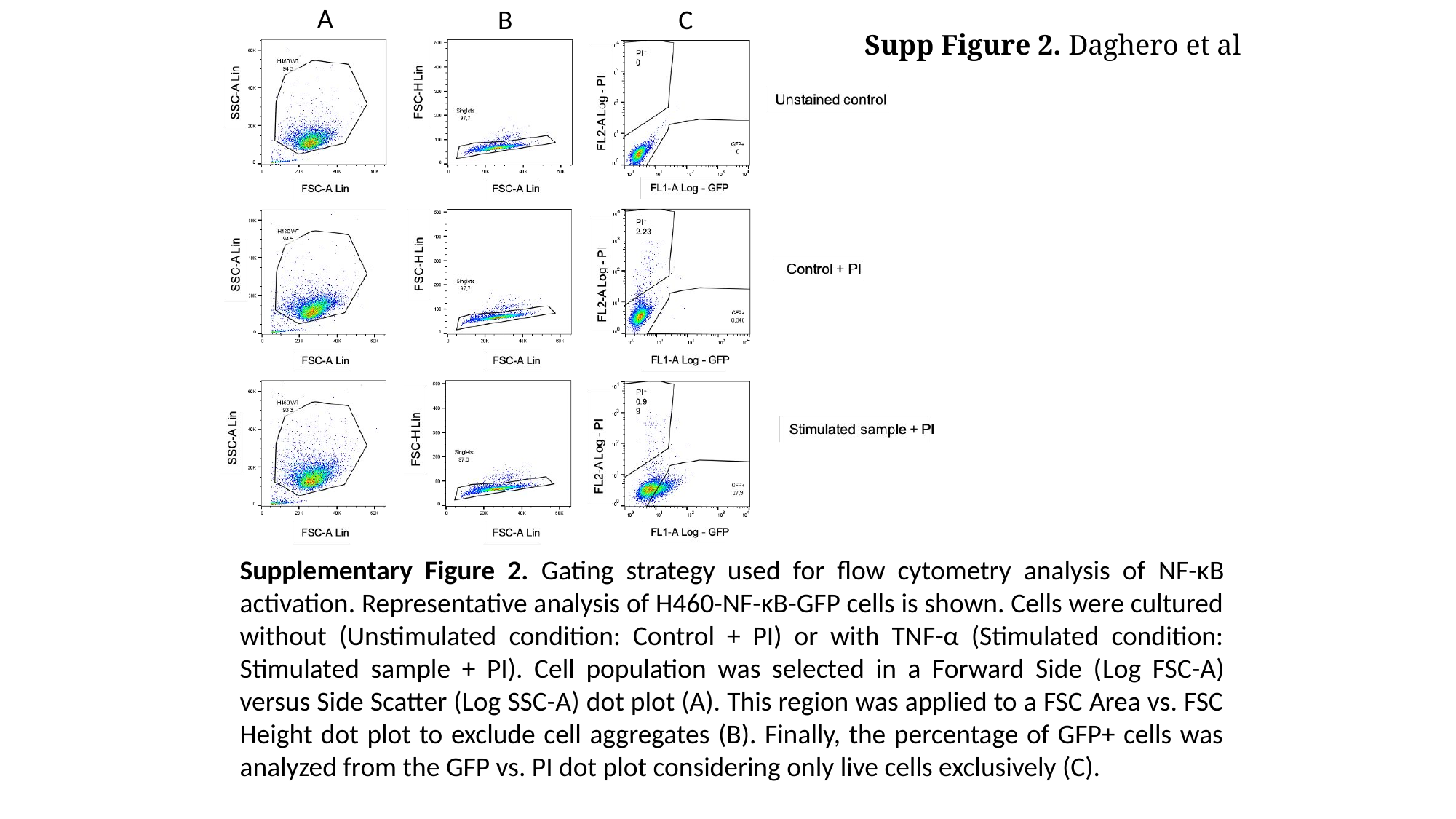

A
B
C
Supp Figure 2. Daghero et al
Supplementary Figure 2. Gating strategy used for flow cytometry analysis of NF-κB activation. Representative analysis of H460-NF-κB-GFP cells is shown. Cells were cultured without (Unstimulated condition: Control + PI) or with TNF-α (Stimulated condition: Stimulated sample + PI). Cell population was selected in a Forward Side (Log FSC-A) versus Side Scatter (Log SSC-A) dot plot (A). This region was applied to a FSC Area vs. FSC Height dot plot to exclude cell aggregates (B). Finally, the percentage of GFP+ cells was analyzed from the GFP vs. PI dot plot considering only live cells exclusively (C).
